# Organizational Factors Are Key Predictors of Physicians’ Confidence in Handling Workplace Violence

**DOI:** 10.3390/healthcare10040637

**Published:** 2022-03-28

**Authors:** Fu-Li Chen, Wen-Hsuan Hou, Jin-Hua Chen, Tao-Hsin Tung, Jeng-Cheng Wu

**Affiliations:** 1 Department of Public Health, College of Medicine, Fu Jen Catholic University, Taipei 24205, Taiwan; 026644@mail.fju.edu.tw; 2 Department of Physical Medicine and Rehabilitation, Taipei Medical University Hospital, Taipei 11031, Taiwan; houwh@tmu.edu.tw; 3 Department of Geriatric and Gerontology, Taipei Medical University Hospital, Taipei 11031, Taiwan; 4 School of Gerontology and Long-Term Care, College of Nursing, Taipei Medical University, Taipei 11031, Taiwan; 5 Cochran Taiwan, Taipei Medical University, Taipei 11031, Taiwan; 6 Graduate Institute of Data Science, College of Management, Taipei Medical University, Taipei 11031, Taiwan; jh_chen@tmu.edu.tw; 7 Institutional Research Center, Office of Data Science, Taipei Medical University, Taipei 11031, Taiwan; 8 Biostatistics Center, Department of Medical Research, Wan Fang Hospital, Taipei Medical University, Taipei 11696, Taiwan; 9 Evidence-Based Medicine Center, Taizhou Hospital of Zhejiang Province, Wenzhou Medical University, Linhai 317000, China; ch2876@gmail.com; 10 Department of Urology, Taipei Medical University Hospital, Taipei 11031, Taiwan; 11 Department of Education, Taipei Medical University Hospital, Taipei 11031, Taiwan; 12 Department of Urology, School of Medicine, College of Medicine, Taipei Medical University, Taipei 11031, Taiwan; 13 Department of Health Promotion and Health Education, College of Education, National Taiwan Normal University, Taipei 10610, Taiwan

**Keywords:** hospital organization, physician, safety, self-confidence, workplace violence

## Abstract

Many studies have investigated health-care workers’ confidence in handling workplace violence with the aim of preventing negative outcomes and fear of such events. The aim of this cross-sectional study was to identify the predictors of physicians’ confidence in handling workplace violence. A self-administered questionnaire was used to collect data on various factors related to workplace violence against physicians in four regional teaching hospitals in northern Taiwan. Of the 180 respondents, 78 (43.3%) had experienced workplace violence in the 3 months preceding the study; they were assigned to the “victim group”. The others (102 respondents) were assigned to the “nonvictim group”. According to multiple linear regression analysis, the factors significantly associated with physicians’ confidence in handling workplace violence in the victim group were perceived organizational support and workplace violence-related training courses. In the nonvictim group, affiliated department and perceived safety climate were key factors. Organizational factors are key predictors of physicians’ confidence in handling workplace violence. Therefore, hospital managers should strive to bolster physicians’ confidence in handling workplace violence. For victims of workplace violence, team-based trainings may improve their interpersonal skills and perceived support from colleagues, both of which can prevent workplace violence events and the repetition of such events.

## 1. Introduction

Workplace violence (WPV) has become a global concern because of its effects on occupational safety and health, especially in health-care settings [1,2,3]. According to data from the 2015 Sixth European Working Conditions Survey, the highest risk of WPV was reported in the health-care sector [2]. Furthermore, Asian and North American countries demonstrated a higher prevalence of WPV than European countries did, according to the results of a 2019 systemic review by Liu et al., and Asian countries have undergone a rising trend in physical violence in the workplace over the past two decades [1].

WPV can negatively affect health-care workers’ physical and psychological well-being and can influence their emotions, social interactions, work performance, and relationships with patients. WPV can further detract from the quality of health-care systems, resulting in a higher social financial burden. Among these potential negative effects, psychological (e.g., depression and anxiety) and emotional (e.g., anger and fear) effects have been demonstrated to be most strongly associated with WPV [4]. These effects can lead to victims being more likely to quit their jobs [5,6]. Although victims often continue to perform their duties after WPV events, such events can negatively affect their work satisfaction and performance [7,8,9,10]. WPV events do not only affect the victims; they can also affect colleagues who witness or are told of such events [11,12]. Health-care workers who perceived their workplace to be a site of WPV were reported to frequently experience fear and suspicion, leading them to become defensive and confrontational and negatively affecting their relationship with patients; this then resulted in an even greater risk of WPV [13]. To minimize the effects of WPV and prevent events and fear of WPV from occurring, studies have investigated health-care workers’ confidence in handling WPV [11]. Because physicians play an essential role in health-care teams, enhancing their confidence in handling WPV is critical to ensuring occupational health and safety.

The etiology of WPV is complex; it varies with personal (e.g., age and sex) [14,15] and organizational (e.g., social support and safety climate) [16,17,18,19] factors, which are associated with different WPV experiences. Safety climate is a critical organizational factor that affects workers’ health; it is reflected in employees’ perceptions of an organization’s respect for safety in the workplace. Organizations can improve their safety climate by ensuring workers’ safety and health through improved safety practices in the workplace [20,21,22]. Employees who work in a more favorable safety climate tend to select active coping strategies to prevent WPV [23], and such strategies are negatively associated with health-care WPV [24,25] and work stress [26]. Social support has also been identified as a preventive factor for WPV in the health-care sector; high perceived social support reduced the severity of the negative effects of experiencing WPV events and employees’ negative attitudes toward their daily work. Furthermore, social support can lead to employees perceiving their work environment to be safer [27,28]. Therefore, Bandura’s Social Cognitive Theory (SCT), focused on learning that occurs in a social context with a dynamic and reciprocal interaction of the person, environment, and behavior factors [29], may provide a conceptual framework to explain the aggressive behavior in children [30] and the workplace [31]. The multifaceted aggregation phenomenon comes from the observational learning of environmental and personal factors which are modulated by beliefs on the positive and negative consequences of behaviors [32]. In addition, the dynamics of SCT between WPV victims and nonvictims may be transmitted by different mechanisms because previous studies on nurses have proposed that the work environment and personal cognition moderate the relationship of direct/indirect WPV and various health outcomes [33,34]. No single factor of WPV should be over-emphasized; WPV should be investigated using a multifactorial perspective. For this reason, intervention from an “integrated organizational perspective” to prevent WPV has been suggested, with training as a key element of such intervention [35]. Organizations must cultivate health-care workers’ abilities to handle WPV to enable these workers to confidently cope with such violence and prevent the incidence of further WPV [20]. Furthermore, health-care providers have become concerned about the increasing incidence of WPV; however, due to low violence reporting rates, evidence to support this concern is lacking [36]. In addition, studies have rarely investigated health-care workers’ confidence in handling WPV, which is a key aspect of health-care workers developing coping behaviors [35] and preventing negative WPV outcomes from occurring [11]. The aim of this study was to evaluate the association between the determining factors of WPV (i.e., safety climate, organizational support, and attendance of training courses) and physicians’ confidence in handling WPV events in hospitals on the basis of the SCT. We also explored the different predictors that existed in victims and non-victims, respectively. The results of this study may enable the development of policies and strategies for preventing and managing WPV against health-care workers.

## 2. Materials and Methods

### 2.1. Participants and Procedures

A total of 401 physicians were recruited from all specialties in four regional teaching hospitals in northern Taiwan (76–113 physicians from each hospital). An anonymous, self-administered questionnaire was distributed to each participant through office mail. Because sample size of at least 180 subjects was recommended based on the rule of thumb “n/30” for a total of 6 independent variables in a regression model [37], our study recruited a total of 189 questionnaires, with an overall response rate of 47.1%. Written informed consent was obtained from all participants, and the study protocols were reviewed and approved by the Human Research Ethics Committee of the participating hospitals.

### 2.2. Measures

The measures in this study comprised four major factors: demographic characteristics, recent WPV experience, and factors associated with confidence in handling WPV, such as safety climate, attendance of training courses, organizational support, and confidence in handling WPV.

### 2.3. Demographic Characteristics

Demographic characteristics included information on the included physicians’ sex, age, and affiliated department. This study followed the approach of previous studies [15,20], and the affiliated department variable was divided into the subcategories of departments with high exposure to WPV (i.e., psychiatry and emergency medicine) and departments with low exposure to WPV (i.e., departments other than psychiatry and emergency medicine).

### 2.4. Experience of WPV in the Preceding 3 Months

Participants were asked to use a binary scale (0 = *no experience* and 1 = *experience*) to indicate whether they had experienced “verbal or physical violence” or “sexual harassment” during their routine work in the 3 months preceding the start of the study. Respondents who indicated having experienced one or both of these types of events were assigned to the “victim group”. Those who reported no experience of either event were assigned to the “nonvictim group”.

### 2.5. Factors Associated with Confidence in Handling WPV

#### 2.5.1. Safety Climate

For safety climate, participating physicians were asked to respond to the items included to evaluate their perceptions of their supervisors’ attitudes toward workplace safety. These items were evaluated on a seven-item scale, modified for application in the study context from an original 16-item questionnaire designed by Zohar and Luria [38], to evaluate group-level (supervisor) perceptions of safety climate. Respondents were asked to indicate whether the management at their hospital “praises workers who pay close attention to safety”, “considers safety when deciding employees’ work pace and schedules”, “discusses methods for improving safety with employees”, “does not disregard safety rules when work is behind schedule”, “emphasizes safety procedures when employees are working under pressure”, “listens carefully to workers’ ideas on improving safety”, and “provides safety training for workers”. Items were scored on a 5-point Likert scale (from 1 = *strongly disagree* to 5 = *strongly agree*). The results of the seven items were summed to represent a safety climate score (ranging from 7 to 35), with a higher score indicating a more favorably perceived safety climate.

#### 2.5.2. Organizational Support

In accordance with details solicited from interviews with health-care workers [39], three items and five response categories (from 1 = *strongly disagree* to 5 = *strongly agree*) were designed to measure the level of organizational support physicians received. The items were: “The other members of my medical team are friendly”, “The other members of my medical team care about me”, and “The other members of my medical team assist me in my medical duties”. The results of the three items were summed to represent an organizational support score (ranging from 3 to 15), with a higher score indicating higher perceived organizational support.

#### 2.5.3. Attendance of Training Courses

Three items were included to assess whether respondents had attended WPV-related training courses in the preceding 3 months on the topics of “protecting oneself from violent behavior”, “management of interpersonal conflict”, and “sexual harassment prevention”. The respondents indicated whether they had attended each type of training by using a binary scale (0 = *did not attend* and 1 = *attended*). The results of the three items were summed to represent a score for WPV-related training (ranging from 0 to 3), with a higher score indicating more favorable training resources.

### 2.6. Confidence in Handling WPV

Two items concerning physicians’ confidence in handling common forms of WPV were evaluated, namely “I am confident in dealing with verbal threats from patients or their relatives” and “I am confident in dealing with verbal or physical sexual harassment from patients or colleagues”. Respondents answered each question on a 5-point Likert scale (from 1 = *extremely unconfident* to 5 = *extremely confident*). The results for these items were summed to represent a confidence in handling WPV score (ranging from 2 to 10), with a higher score indicating a higher confidence level.

### 2.7. Reliability and Validity

The survey questionnaire was developed by a panel of experts and was reviewed by three hospital clinicians and two occupational health professionals to ensure its construct validity. To verify the questionnaire’s validity and reliability, it was piloted in a convenience sample from a public hospital in Taipei City. The results demonstrated that the questionnaire had satisfactory validity and internal consistency with respect to its confidence in handling WPV, safety climate, and organizational support scales; the Cronbach’s alphas for these three measures for the formal study were 0.819, 0.928, and 0.936, respectively, indicating good reliability.

### 2.8. Data Analysis

Statistical analysis was performed using PASW Statistics Version 18.0 (SPSS, Chicago, IL, USA). The descriptive results of the categorical variables, such as the physicians’ sex, affiliated department, and WPV experience in the preceding 3 months, were expressed as numbers and percentages for each category. Continuous variables, such as the physicians’ age, confidence in handing WPV, and related organizational factors (safety climate, organizational support, and attendance of training courses), were expressed as the mean ± standard deviation (SD). The chi-squared test was adopted to assess differences in categorical variables (sex and affiliated department), and a two-sample *t* test was used to assess differences in continuous variables (age, confidence in handling WPV, safety climate, level of organizational support, and attendance of training courses) between the victim and nonvictim groups. For univariate analysis of each study group, a two-sample *t* test was adopted to assess differences in the mean values of confidence in handling WPV for each category of physicians’ demographics. A Pearson correlation test was conducted to examine the association between physicians’ confidence in handling WPV and associated factors (age, safety climate, level of organizational support, and attendance of training courses). Finally, multiple linear regressions, controlling for sex, age, and department affiliation, were used to investigate the independence of environmental factors (i.e., safety climate and organizational support) and the personal factor (i.e., attendance of training courses) in the organization associated with physicians’ confidence in handling WPV between victim and nonvictim groups. Missing the key question of the experience of WPV was excluded during descriptive analysis, and respondents with missing information for any of the covariates were also automatically excluded in the multiple regression analyses [40]. A *p*-value of <0.05 was considered to represent a significant difference.

## 3. Results

The descriptive results for respondent demographics, experience of WPV in the preceding 3 months, confidence in handling WPV, and associated factors are listed in Table 1. Nine of the respondents who did not answer our key question about the experience of WPV were excluded. A total of 180 respondents were with a mean age of 43.0 years (SD = 9.0); of the respondents, 73.9% were men, and 15.0% were affiliated with departments with high exposure to WPV (psychiatry or emergency medicine). The mean score for their confidence in handling WPV was 7.1 (SD = 1.5), which was between fair and confident (average of 3.6 on the 5-point Likert scale). The mean scores for perceived safety climate and organizational support were 24.0 (SD = 4.6) and 11.5 (SD = 1.9), respectively. Both were near-median levels. The mean score for the physician training rating was 0.3 (SD = 0.6), meaning that participants attended fewer than one of the three listed types of WPV-related courses in the 3 months preceding the survey. Seventy-eight respondents (43.3%) had experienced at least one event of WPV during their routine work in the 3 months preceding the study (75 physicians had experienced verbal or physical violence and 18 had experienced sexual harassment). Notably, 15 (83.3%) of the 18 sexual harassment victims had experienced verbal or physical violence simultaneously. These 78 were assigned to the “victim group”; the 102 respondents who did not experience these forms of WPV in the same period were assigned to the “nonvictim group”. Physicians affiliated with psychiatry or emergency medicine departments had a higher incidence of being a victim of WPV, and the victim group had a significantly lower level of perceived safety climate than the nonvictim group did (22.8 vs. 25.0, *p* < 0.01).

Univariate analysis using a *t* test for categorical variables (sex and affiliated department) and Pearson’s correlation coefficient for continuous variables were used to explore the correlation between physicians’ confidence in handling WPV and associated factors. The results are presented in Table 2. Among participants, respondents who were male, older, and affiliated with psychiatry or emergency medicine departments had significantly higher levels of confidence in handing WPV. All three organizational factors (perceived safety climate, organizational support, and training resources) had significant positive correlations with physicians’ confidence in handling WPV. With the exception of the physicians’ affiliated department, all of the listed variables were significant associated factors in the victim group. By contrast, only the respondents’ affiliated departments and perceived safety climate were significant associated factors in the nonvictim group.

To predict the factors related to physicians’ confidence in handling WPV, multiple liner regression was conducted. The results presented in Table 3 reveal that affiliated department (β = 0.66, 95% CI = 0.06–1.25), perceived safety climate (β = 0.09, 95% CI = 0.04–0.14), perceived organizational support (β = 0.12, 95% CI = 0.01–0.25), and attending training courses (β = 0.41, 95% CI = 0.07–0.75) were significant predictors for all participants (adjusted R^2^ = 0.206, *p* < 0.001). For the victim group, two organizational factors, namely perceived organizational support (β = 0.30, 95% CI = 0.13–0.46) and attendance of training courses (β = 0.58, 95% CI = 0.04–1.12), were identified as significant predictors (adjusted R^2^ = 0.313, *p* < 0.001). In the nonvictim group, respondents’ affiliated department (β = 1.94, 95% CI = 0.05–3.82) and perceived safety climate (β = 0.13, 95% CI = 0.06–0.20) were identified as significant predictors (adjusted R^2^ = 0.194, *p* < 0.001).

## 4. Discussion

Our study revealed that more than 40% (78 physicians) of the 180 respondents experienced work-related verbal or physical threats (75 victims) or sexual harassment (18 victims) in the 3 months preceding the study, and more than 80% of the victims of sexual harassment also experienced verbal or physical threats during the same period. These results are representative of the global problem of WPV against health-care workers and indicate that WPV is a severe occupational hazard for Taiwanese physicians. Because many forms of WPV often co-occur [41], hospital managers should assist the victims of WPV in comprehensively coping with WPV rather than focusing only on the WPV event.

This study was conducted to investigate the predictive factors of physicians’ confidence in handling WPV through self-administered questionnaires. None of the physicians’ demographic characteristics were significantly associated with their confidence in handling WPV, with the exception of their affiliated department. Respondents affiliated with high-WPV-exposure departments (psychiatry and emergency medicine) had a higher level of confidence in handing WPV situations. Further studies are required to investigate whether the more favorable safety climate in the psychiatry and emergency medicine departments results from professional training and support from colleagues in coping with WPV. Physicians who have low confidence in handling WPV may leave a workplace if they perceive it as having an unfavorable safety climate, which may result in a shortage of physicians in departments with high exposure to WPV. If a physician is confident in handing WPV, they are less likely to fear the workplace and are more likely to unwaveringly perform their job duties.

In this study, all of the included organizational factors were significantly associated with physicians’ confidence in handling WPV. Physicians’ perceived safety climate was the strongest predictor of their confidence in handling WPV. All employees have the right to work in a safe environment without the threat of violence; furthermore, employers have the responsibility of ensuring employees’ safety in the workplace. The concept of a safety climate was introduced in the health-care context in the late 1990s. This concept has since been demonstrated to be positively related to safety practices that ensure health-care workers’ occupational safety [42,43,44,45]. Safety climate is a key organizational factor affecting workers’ health, and it ensures workers’ safety and health by improving their compliance with safety practices in the workplace. However, safety climates are often perceived to be worse in Taiwan than they are in many Western countries [26,46]. For WPV in the context of health-care services, more favorably perceived safety climates have been demonstrated to be significantly correlated with a decrease in the incidence of WPV events [18,19,26,47]. Furthermore, perceived safety climate was reported to be a protective factor against WPV that mediates the relationship between WPV and its negative effects, job satisfaction, and work engagement [48]. For confidence in handling WPV, an employee’s perceived safety climate with respect to their perception of their employer’s prevention of violence was significantly and positively correlated with their ability to handle WPV, which reduced their fear of future violence and prevented negative outcomes from WPV events [49]. Therefore, researchers have suggested that employers cultivating a safety climate with a zero-tolerance policy for WPV is essential in eliminating WPV in the health sector [14,50]; however, few studies have investigated the association between safety climate and physicians’ confidence in handling WPV.

For physicians who had recently experienced WPV, we found that perceived organizational support and attendance of training courses were more positively associated with their confidence in handling WPV than their perceived safety climate. According to Bandura’s SCT, confidence is a key proximal predictor of developing behaviors required to manage prospective situations [29]. When individuals encounter challenges, those with higher confidence levels are more likely to believe themselves capable of performing well in managing the challenge and less likely to run away from it. Evaluations of WPV-related training in the health-care sector have demonstrated that confidence is a key influencing factor in the development of safety behaviors after training [35]. Therefore, establishing policies that improve physicians’ confidence in handing threats of violence or assault are essential in avoiding WPV and the potential repetition of such events. In this study, preventive WPV skills training significantly improved physicians’ confidence in handling WPV after adjustment for confounding factors (i.e., sex, age, and department affiliation). Most notably, a previous systematic review has proposed team-based interactive training workshops not only improved physicians’ interpersonal skills but also led to higher perceived support from colleagues, both of which can prevent the risk and repetition of WPV events [51].

Our study results also reflect that the occurrence of WPV was based on the theoretical framework of Badura’s SCT; the environmental factors such as safety climate and organizational support in accordance with the personal factor of attendance of training courses could significantly affect the violent behavior or the confidence in handling WPV. Thus, health care requires professional teamwork including physicians, nurses, assistants, technicians, and administrative personnel; perceived social support from management and colleagues is essential for health-care workers to develop both professionalism and coping mechanisms when handling aggressive patients [52]. Social support has been identified as a protective factor against WPV in the health-care sector; it reduced the occurrence of negative outcomes from WPV events and employees’ development of negative attitudes toward their work [27,28]. Social support also leads employees to perceive their working environment as safer, and victims of WPV have higher confidence in their ability to handle WPV situations [53], which is similar to our study results that the provision of organization support and training courses would significantly increase the victim’s confidence in handling WPV. Differently, organizational factors related to a better safe climate and higher exposure of WPV would increase nonvictims’ confidence in handling WPV, which could be explained by the phenomena of healthy organizational culture [54] and healthy worker effects [8]. Thus, the WHO’s guideline for WPV in the health sector has stated prevention plans and response strategies separately [14].

### 4.1. Limitation

This study has several limitations. First, all participants were recruited from one of four regional teaching hospitals in northern Taiwan, which may hinder the external validity and generalizability of the results. Because WPV varies with cultural differences, caution should be exercised when applying these results to hospitals of different levels or located in different regions. The generalizability of the results must be verified through further investigations of physicians’ workplaces. Second, the convenience sample with a lower than 50% response rate may contain selection bias. However, the sample size and statistical power have been evaluated as being sufficient. Furthermore, the 95% CIs were relatively narrow, supporting the reliability of our findings. Third, the results were obtained from a cross-sectional study; further longitudinal follow-up studies should be conducted to evaluate the causal relationships among the determining factors and physicians’ confidence in handling WPV. In addition, although we have adjusted for several sociodemographic variables (i.e., sex, age, and department affiliation) in multiple linear regression models, several personal characteristics (e.g., educational level and number of work years) and environmental factors (e.g., work experience and union membership) may be potential confounders. Moreover, because the etiology and context of sexual harassment (sexual overtures) and sexual violence could be different [55], different types of WPV and victim’s sex have different impacts for people’s self-efficacy of handing WPV and result in different strategies of WPV prevention [56]. Further study would be needed to explore the relationship of physician’s confidence stratified by sex or type of aggression; for example, the underlying mechanism for the co-occurrence of sexual harassment and physical violence behaviors in the workplace [41].

### 4.2. Practical Implementation

Our study could provide not only the employees themselves, but also hospital managers with responsibility for building physicians’ confidence in handling WPV and implement strategies to assist victims of WPV. Because physicians play a key role in health-care teams, building their confidence in handling WPV will enable them to set an example for other team members. Further WPV prevention programs could be implemented according to the framework of SCT to promote a healthy working environment.

## 5. Conclusions

In this study, physicians’ demographic characteristics (age and sex) were not significantly associated with their confidence in handling WPV. However, all of the included organizational factors were significant predictors of the physicians’ confidence in handling WPV; furthermore, our results revealed different associations for victims and nonvictims of WPV. Rather than the employees themselves, hospital managers should be responsible for building physicians’ confidence in handling WPV; they must implement strategies to assist victims of WPV. Because physicians play a key role in health-care teams, building their confidence in handling WPV will enable them to set an example for other team members. Therefore, hospital management and relevant government agencies should establish a zero-tolerance policy toward WPV; these authorities should further be responsible for providing health-care workers with sufficient support systems to prevent WPV events and repeated events from occurring.

## Figures and Tables

**Table 1:** Descriptive information of respondents’ demographics, experience of WPV ^a^ in the preceding 3 months, confidence in handling WPV, and associated factors.

	Total, N = 180			Victim, N = 78			Nonvictim, N = 102			
Variables (Range)	N	%	Mean ± SD	N	%	Mean ± SD	N	%	Mean ± SD	*p*-Value
Sex ^c^										0.09
Male	133	73.9		53	67.9		80	78.4		
Female	46	25.6		25	32.1		21	20.6		
Age (years)			43.0 ± 9.0			42.4 ± 8.6			43.5 ± 9.3	0.43
Department ^b,c^										<0.01
High exposure to WPV	27	15.0		24	30.8		3	2.9		
Low exposure to WPV	152	84.4		53	67.9		99	97.1		
Safety climate (7–35)			24.0 ± 4.6			22.8 ± 2.4			25.0 ± 5.5	<0.01
Organizational support (3–15)			11.5 ± 1.9			11.4 ± 1.1			11.6 ± 1.7	0.51
Attendance of training courses (0–3)			0.3 ± 0.6			0.3 ± 0.6			0.4 ± 0.7	0.82
Confidence in handling WPV (2–10)			7.1 ± 0.5			7.0 ± 0.6			7.2 ± 0.5	0.47

^a^ WPV = workplace violence. ^b^ Department with high exposure to WPV: departments of psychiatry or emergency medicine; Department with low exposure to WPV: departments other than psychiatry and emergency medicine. ^c^ Total percentages do not add up to 100 due to missing information.

**Table 2:** Univariate analysis of physicians’ confidence in handling WPV ^a^ and associated factors.

	Total, N = 180			Victim, N = 78			Nonvictim, N = 102		
Variables	Mean ± SD	Pearson r	*p*	Mean ± SD	Pearson r	*p*	Mean ± SD	Pearson r	*p*
Sex									
Male	7.3 ± 1.6		0.009	7.3 ± 1.8		0.025	7.3 ± 1.5		0.209
Female	6.6 ± 1.2			6.4 ± 1.2			6.9 ± 1.2		
Age (years)		0.23	0.003		0.28	0.014		0.18	0.078
Department ^b^									
High exposure to WPV	7.7 ± 1.6		0.037	7.5 ± 1.6		0.070	9.0 ± 1.0		0.031
Low exposure to WPV	7.0 ± 1.5			6.8 ± 1.6			7.2 ± 1.5		
Safety climate		0.31	<0.001		0.24	0.034		0.36	<0.001
Organizational support		0.25	0.001		0.41	<0.001		0.07	0.481
Attendance of training courses		0.22	0.004		0.25	0.030		0.20	0.052

^a^ WPV= workplace violence. ^b^ Department with high exposure to WPV: departments of psychiatry or emergency medicine; Department with low exposure to WPV: departments other than psychiatry and emergency medicine.

**Table 3:** Multiple liner regression of physicians’ confidence in handling WPV ^a^ and associated factors.

	Total, N = 169		Victim, N = 75		Nonvictim, N = 94	
Variables	β	95% CI	β	95% CI	β	95% CI
Constant	2.36		0.29		4.49	
Sex (male vs. female)	0.36	−0.13 to 0.85	0.50	−0.22 to 1.22	0.39	−0.31 to 1.10
Age (years)	0.01	−0.01 to 0.04	0.03	−0.01 to 0.07	0.01	−0.02 to 0.04
Department ^b^ (high vs. low)	0.66 *	0.06 to 1.25	0.47	−0.23 to 1.17	1.94 *	0.05 to 3.82
Safety climate	0.09 ***	0.04 to 0.14	0.06	−0.02 to 0.14	0.13 ***	0.06 to 0.20
Organizational support	0.12 *	0.01 to 0.25	0.30 **	0.13 to 0.46	−0.14	−0.33 to 0.05
Attendance of training courses	0.41 *	0.07 to 0.75	0.58 *	0.04 to 1.12	0.25	−0.20 to 0.69
F	8.568		6.617		4.722	
*p*	<0.001		<0.001		<0.001	
R	0.483		0.607		0.496	
R^2^	0.233		0.369		0.246	
Adjusted R^2^	0.206		0.313		0.194	

^a^ WPV = workplace violence. ^b^ Department with high exposure to WPV: departments of psychiatry or emergency medicine; Department with low exposure to WPV: departments other than psychiatry and emergency medicine. Adjusted for sex (male vs. female), age (years), and department (high vs. low). * *p* < 0.05, ** *p* < 0.01, *** *p* < 0.001.

## Data Availability

The datasets generated and analyzed during the study are not publicly available to preserve the participants’ anonymity. Requests for the data can be made to the corresponding author.
